# Pathogenesis of oral FIV infection

**DOI:** 10.1371/journal.pone.0185138

**Published:** 2017-09-21

**Authors:** Craig Miller, Karen Boegler, Scott Carver, Martha MacMillan, Helle Bielefeldt-Ohmann, Sue VandeWoude

**Affiliations:** 1 Department of Microbiology, Immunology, and Pathology, Colorado State University, Fort Collins, Colorado; 2 School of Zoology, University of Tasmania, Hobart, Tasmania, Australia; 3 Australian Infectious Diseases Research Centre, University of Queensland, St. Lucia, Queensland, Australia; Centers for Disease Control and Prevention, UNITED STATES

## Abstract

Feline immunodeficiency virus (FIV) is the feline analogue of human immunodeficiency virus (HIV) and features many hallmarks of HIV infection and pathogenesis, including the development of concurrent oral lesions. While HIV is typically transmitted via parenteral transmucosal contact, recent studies prove that oral transmission can occur, and that saliva from infected individuals contains significant amounts of HIV RNA and DNA. While it is accepted that FIV is primarily transmitted by biting, few studies have evaluated FIV oral infection kinetics and transmission mechanisms over the last 20 years. Modern quantitative analyses applied to natural FIV oral infection could significantly further our understanding of lentiviral oral disease and transmission. We therefore characterized FIV salivary viral kinetics and antibody secretions to more fully document oral viral pathogenesis. Our results demonstrate that: (i) saliva of FIV-infected cats contains infectious virus particles, FIV viral RNA at levels equivalent to circulation, and lower but significant amounts of FIV proviral DNA; (ii) the ratio of FIV RNA to DNA is significantly higher in saliva than in circulation; (iii) FIV viral load in oral lymphoid tissues (tonsil, lymph nodes) is significantly higher than mucosal tissues (buccal mucosa, salivary gland, tongue); (iv) salivary IgG antibodies increase significantly over time in FIV-infected cats, while salivary IgA levels remain static; and, (v) saliva from naïve Specific Pathogen Free cats inhibits FIV growth *in vitro*. Collectively, these results suggest that oral lymphoid tissues serve as a site for enhanced FIV replication, resulting in accumulation of FIV particles and FIV-infected cells in saliva. Failure to induce a virus-specific oral mucosal antibody response, and/or viral capability to overcome inhibitory components in saliva may perpetuate chronic oral cavity infection. Based upon these findings, we propose a model of oral FIV pathogenesis and suggest alternative diagnostic modalities and translational approaches to study oral HIV infection.

## Introduction

Feline immunodeficiency virus (FIV) is a naturally occurring lentivirus of domestic cats and non-domestic feline species that is genetically, structurally, and biochemically similar to human immunodeficiency virus (HIV), thereby providing a valuable animal model for studying HIV infection [1–6]. While HIV transmission typically occurs via parenteral or transmucosal venereal contact, oral transmission of HIV via receptive oral intercourse, breast feeding, and human bites has been well-documented, especially in the presence of a disrupted oral mucosal barrier (ie: epithelial ulceration), inflammation, and/or concurrent oral infections [7–15]. These alternative modes of HIV transmission represent a significant health concern to at-risk individuals, including recipients of oral intercourse, infants of HIV-positive mothers, and dental health professionals. Additionally, recent studies have shown that both HIV viral RNA and proviral DNA are detectable in saliva of infected individuals, and salivary RNA levels are correlated with levels in circulation; facts that have been largely overlooked in studies of HIV pathogenesis [16, 17]. Furthermore, HIV antibodies in saliva may lend themselves to noninvasive diagnostic methodologies via rapid detection of saliva specific antibodies, and HIV therapies that incorporate antiviral properties of saliva have been considered, including the use of inhibitory molecules as components of commercial lubricants [14, 18–23].

HIV, Simian Immunodeficiency Virus (SIV), and FIV have all been shown to target lymphocytes of the alimentary tract early during viral infection, resulting in long term consequences for mucosal immune function [24–28]. Several studies have linked lentiviral-induced gastrointestinal mucosal insult to chronic immune activation associated with infection via translocation of bacteria or bacterial antigens [25, 29–32]. It is feasible that the oral cavity, with its rich lymphoid tonsillar structures, is similarly impacted by lentiviral infection, and may serve as important reservoir for lentiviral persistence [27]. Unlike primate lentiviral infections, which are primarily considered to be transmitted venereally, during maternal-offspring interactions, or via exchange of blood products, FIV is believed to be primarily transmitted from cat to cat via bite wounds during antagonistic or mating interactions. However, vertical transmission of FIV has been experimentally shown via colostrum and milk, and may provide an appropriate model to study mother-to-offspring transmission of HIV [33, 34]. Additionally, FIV periodontitis/gingivitis is a hallmark of both naturally occurring and experimental FIV infections, and characteristic lesions of FIV gingivitis are similar to those frequently reported for HIV-associated dental disease, such as linear bands of erythema along the gingival margin and ulcerative to necrotizing lesions within the gingival and buccal mucosa [1, 6, 35–44]. Despite the fact that FIV associated gingival disease is widely diagnosed, very few studies have assessed mechanisms of FIV salivary excretion, transmission, and pathogenesis [42, 45]. Further, recent reports document that FIV infection is poorly or rarely transmitted among cats in multi-cat households, calling into question whether FIV salivary transmission is limited by innate immune barriers in stable social groups [46].

Studies of FIV salivary transmission and pathogenesis of oral disease have the potential to reveal sites of lentiviral replication and persistence, mechanisms of salivary excretion, and oral lentiviral immune responses and diseases—thereby expanding our knowledge of the pathogenesis of FIV disease and mechanisms of orally transmitted pathogens. Although it is widely accepted that FIV is primarily transmitted by biting, and much less efficiently during social grooming, few studies have evaluated FIV oral infection kinetics and transmission mechanisms over the last 20 years. Modern quantitative analyses applied to natural FIV oral infection could significantly further our understanding of the temporal events that occur during FIV oral infection pathogenesis, and may thus aid in the design of improved diagnostics, vaccines and vaccine modalities, choice of adjuvants, and design and delivery of antiviral agents for FIV. As salivary excretion and kinetics of HIV infection is also understudied, identification of similarities and differences between FIV and HIV oral pathogenesis can also ultimately enhance our understanding of HIV-associated oral disease.

Previous studies of FIV oral transmission and pathogenesis have sought to characterize viral excretion mechanisms by detection of saliva FIV RNA, DNA or antibodies, or though isolation of virus in saliva or oral tissues [42, 44, 45, 47, 48]. However, because much of this work was performed before the development of quantitative analyses, there has been limited understanding of the temporal events that take place in the oral cavity during FIV infection. To address these knowledge gaps, we opportunistically evaluated salivary transmission characteristics in cats intravenously inoculated with a well-characterized immunopathogenic strain of FIV (FIV_C36_) [36, 49]. Blood, saliva, and tissue samples from 18 cats enrolled in a clinical trial were evaluated for viral RNA and proviral DNA by real-time quantitative PCR (qPCR) analysis, in addition to other clinical and hematologic parameters associated with disease progression. Additionally, quantification of total IgA/IgG and FIV-specific IgA/IgG antibodies in saliva was performed using microsphere immunoassay (MIA), and histologic changes in tissues were assessed. Saliva from naïve SPF (specific pathogen free) cats was incubated with a viral stock of FIV_C36_ and tested for capacity to inhibit viral growth in Crandall feline kidney (CRFK) cell cultures. Coincident studies evaluating hematologic and clinical features of infection in these same cats allowed comparisons of oral and peripheral characteristics of viral infection [50, 51].

This study represents the first quantitative analysis of FIV RNA and DNA in saliva and oral tissues of FIV-infected cats, evaluated in conjunction with quantification of anti-FIV IgG and IgA antibodies in saliva and assessment of concurrent alterations in the oral mucosa to provide a comprehensive assessment of the pathogenic events that contribute to the development of oral FIV infection. Overall, our findings characterize sites of FIV replication and persistence, and reveal that oral cavity viral kinetics and antibody responses mirror that of the periphery; suggesting that immunological and tissue level barriers are ineffective at inhibiting FIV viral replication and excretion at the oral mucosal surface.

## Materials and methods

### Ethics statement

This study was approved by the Colorado State University Institutional Animal Care and Use Committee; 09-064A-01—New Therapies for Retroviral Diseases. Colorado State University's animal care program is licensed by the United States Department of Agriculture (USDA), accredited by Association for Assessment and Accreditation of Laboratory Animal Care (AAALA) International, and holds an Office of Laboratory Animal Welfare (OLAW) assurance (A3572-01). In accordance with the approved IACUC protocol, if any animal exhibited significant clinical abnormalities (vomiting, diarrhea, lethargy, refusal of food > 24 hours, labored breathing, dehydration, >5% weight loss from one week to the next), a complete physical exam with additional bloodwork was performed. In the case that a study animal exhibited severe discomfort or symptoms listed above which worsened and did not respond to symptomatic therapy, humane euthanasia was to be performed and followed by a complete necropsy. Time points for euthanasia of study animals was determined by previously established time intervals to assess acute FIV infection, or at the discretion of a clinical veterinarian based upon the severity of any associated clinical signs and/or response to symptomatic therapy. Humane euthanasia procedures were conducted by phenobarbital overdose in accordance with IACUC protocols and American Veterinary Medical Association (AVMA) Guidelines for the Euthanasia of Animals. Prior to euthanasia, all study animals were anesthetized by intramuscular injection of ketamine (20mg/kg) and acepromazine (2mg/kg) to minimize animal suffering and distress. No animals died without euthanasia during this study as a result of experimental procedures. All study animals were monitored daily by animal care personnel for development of clinical signs of FIV infection. Any animals exhibiting clinical signs either associated with FIV infection or other untoward condition were evaluated and treated as prescribed by the clinical veterinarian. One animal developed clinical signs at week 6 post-infection, and was humanely euthanized at the discretion of the attending veterinarian. All other animals were euthanized at the end of the experimental study.

### *In vivo* protocol

Twenty-four, 8–11 week old, specific pathogen free (SPF) cats, procured from Cedar River Laboratories, Mason City, IA, and the Andrea D. Lauerman Specific Pathogen Free Feline Research Colony, Fort Collins, CO, were housed within barrier rooms in accordance with Colorado State University (CSU) IACUC-approved protocols at a CSU AAALAC-international accredited animal facility. All animals were part of an anti-retroviral protocol, and were acclimated to the facility for 2 weeks prior to initiation of the study. At day 0, eighteen cats were intravenously inoculated with 1ml of a 1:10 dilution of a previously characterized FIV_C36_ viral stock that is acutely immunopathogenic and induces reproducible high titer viremia [37, 49]. Six additional cats were sham inoculated as negative controls. Over the course of the study, 12 of the FIV-infected cats received experimental anti-retroviral treatment, while 6 FIV-infected cats and the 6 sham-inoculated cats received no anti-retroviral treatment and served as positive and negative controls, respectively.

### Evaluation of viral RNA and DNA in blood and saliva

Blood and saliva samples were collected from cats at 7-day intervals, beginning at 15 days post-infection and ending at 92 days post-infection. Blood samples were obtained as previously described [50]. Saliva was obtained from under the tongue and cheek pouches of each cat using sterile cotton swabs, which were immediately broken off into 1.5ml microcentrifuge tubes containing 200μL of RNAlater Solution (Ambion, Austin, TX) and stored at -20°C. At processing, stored swabs were thawed at room temperature, vortexed vigorously for 1 min, and centrifuged at 400 x g for 1 min. To collect saliva from the swab tip, swabs were inverted using sterile forceps, placed back into microcentrifuge tubes, spun at 2000 rpm for 2 min, and then discarded, leaving the saliva/RNAlater solution in the microcentrifuge tube.

Viral RNA was extracted from saliva using an RNAqueous total RNA isolation kit (Ambion, Austin, TX), according to manufacturer’s instructions. Samples were eluted in 50μL and ethanol precipitated overnight at -20°C (2.5 vol 100% ethanol, 0.1 vol 3M sodium acetate, and 1.0μL glycogen). Precipitated RNA was pelleted at 18,000 x g for 20 min at 4°C and re-suspended in 20μL of RNA Storage Solution (Ambion, Austin, TX). RNA from each sample was converted to cDNA using the RETROscript reverse transcription kit (Ambion, Austin, TX). The total volume of extracted RNA was transferred into two 20μL reactions and converted using random decamer primers and following manufacturer’s instructions for reverse transcription without heat denaturation of RNA. FIV-C was detected by qPCR in triplicate using an iQ5 thermocycler (Bio-Rad, Hercules, CA) with reaction components, cycling parameters, and FIV-C primers and probes as previously described [52, 53]. To quantitate viral copy number in each reaction, a six-point standard curve was generated by diluting FIV-C virus stock in a 10-fold dilution series into RNAlater solution. Each dilution was then extracted and converted to cDNA as described above, and assigned a copy number value based on comparison to a FIV C gag plasmid standard curve ranging from 10^5^ to 10^−1^ copies per reaction. A Ct threshold was set according to the run data for each plate, and Ct values greater than those of negative controls were included in the analysis. The resulting copy number data for each sample was analyzed using Prism 4 (GraphPad Software, La Jolla, CA). Triplicate values for each sample were averaged, and calculated to determine viral copies per mL saliva. Standard error was calculated for each treatment group at each time-point. Viral RNA was extracted from blood and quantified by qPCR as previously described [50].

Proviral DNA was extracted from saliva of all cats for time-points 43 and 64 days post-inoculation using a DNeasy Blood and Tissue Kit (Qiagen, Valencia, CA) and a user-adapted protocol for purification of total DNA from animal saliva [54]. Samples were eluted in 100μL, and then ethanol precipitated (2.5 vol 100% ethanol, 0.1 vol 3M sodium acetate, and 1.0μL glycogen) overnight at -20°C. Precipitated DNA was then pelleted at 18,000 x g for 20 min at 4°C and re-suspended in 20μL H_2_O. FIV-C provirus was quantitated by qPCR in duplicate using an iQ5 thermocycler (Bio-Rad, Hercules, CA), FIV-C primers and probe, and FIV-C plasmid standards as previously described [52, 53]. Proviral copy number was then normalized to copies per 10^6^ cells by quantitating genomic DNA using real-time qPCR targeted to feline GAPDH. A GAPDH plasmid standard curve was prepared as previously described [55] and the following primers were used for qPCR: GAPDH-F forward primer, 5’- AAGGCTGAGAACGGGAAAC -3’; GAPDH-R reverse primer, 5’- CATTTGATGTTGGCGGGATC– 3’. GAPDH qPCR for each sample was set up in 25 μl using the following reaction components: 2 μl sample DNA, 8.5 μl de-ionized water, 1 μl GAPDH-F, 1 μl GAPDH-R, and 12.5 μl SsoFast EvaGreen Supermix (Bio-Rad, Hercules, CA). Reaction mixtures were denatured at 95°C for 30 seconds and then followed by a two-step reaction cycle protocol of 40 cycles that alternated between 95°C for 5 seconds and 60°C for 10 seconds. Proviral DNA was extracted from blood, quantified by qPCR, and normalized to a GAPDH standard curve to determine the number of cell equivalents per DNA sample. Proviral copy number per cell was calculated as previously described [49, 50].

To confirm the presence of infectious FIV particles in saliva of infected cats, duplicate cell cultures consisting of GFox cells (CrFK cells overexpressing CD134) were established in 96-well plates at 20,000 cells/well and allowed to attach at 37°C overnight [53, 56]. GFox cell cultures were maintained at 37°C and 5% CO_2_, in 240 μl of culture medium composed of Dulbecco's modified Eagle's medium (DMEM) with GlutaMAX-1, 10% fetal bovine serum (FBS), and 1x penicillin-streptomycin (10,000 U/liter penicillin and 10,000 μg/liter streptomycin), as well as 1 μg/ml of Fungizone^®^ (Amphotericin B; Life Technologies) [57]. At day 0, 10 μl of saliva from 5 FIV-infected and 1 sham-inoculated (uninfected) cats (collected as previously described) were added to the previously established Gfox cell culture, bringing the total volume to 250 μl (1:25 dilution of saliva). A parallel set of wells containing Gfox cells without virus was incubated with media only (negative control). Cell cultures were then incubated at 37°C for 12 hours, at which point all culture media was removed from each well, discarded, and replaced with 250 μl of fresh culture media. GFox cells were visually inspected at days 7, 10, 14, 21, and 28 post-inoculation by inverted light microscopy for evidence of cell growth, attachment, syncytial cell formation, detachment, and cell death. At days 7, 14, 21 and 28 post-inoculation, 125 μl of supernatant was removed from each well, frozen at -80°C, and replaced with 125 μl of fresh culture media. The supernatant collected from each well and each time point was then assayed for the detection of FIV p26 antigen by capture ELISA using previously described protocols [58]. For each treatment, absorbance (Abs) was measured at 450 nm and a background threshold value was established by calculating the mean Abs of FIV-negative (naïve) saliva plus 3 standard deviations. All Abs values above this threshold were considered positive.

### Evaluation of viral RNA and DNA in oral tissues

DNA was extracted from frozen necropsy tissues using a DNeasy Blood and Tissue Kit (Qiagen, Valencia, CA), and eluted in 200μl AE buffer. All samples were adjusted to 20ng/μl using 1X TE buffer and FIV-C provirus was quantitated by qPCR using previously described reaction components, cycling parameters, and FIV-C primers and probes [52, 53]. Proviral copy number within these tissues was quantitated using a standard curve with 1:10 serial dilutions of FIV-C gag plasmid into 1X TE buffer, ranging from 10^5^ copies to 10^1^ copies per reaction. Resulting proviral copy numbers were normalized to copies per 10^6^ cells based on the total amount of DNA present in the reaction (100ng) as previously described [49].

RNA was extracted from frozen necropsy tissues using an RNeasy Mini Kit (Qiagen, Valencia, CA) and tissue homogenizer (MP Biomedicals, Solon, OH). Superscript II (Invitrogen), random primers (Invitrogen), and RNase Out (Invitrogen) were used to synthesize cDNA by reverse transcription. Real-time qPCR quantification of viral RNA was then performed on an iQ5 thermocycler (Bio-Rad, Hercules, CA) using previously described reaction components, cycling parameters, and FIV-C primers and probes [52, 53]. Viral RNA was normalized to GAPDH expression as previously described [46] using the ΔΔCT method [59].

### Histological evaluation

Necropsy was performed on sixteen FIV-inoculated cats at 92 days post inoculation; one sham-inoculated cat was coincidently necropsied to provide FIV negative control tissues. Palatine tonsils, retropharyngeal lymph nodes, submandibular salivary glands, tongue and buccal mucosa were collected. Necropsy tissues were then halved and placed into either 1ml tubes and frozen at -80°C, or into standard tissue cassettes that were then fixed in 10% neutral-buffered formaldehyde for 24 hours prior to trimming and processing for histology. Five μm paraffin sections were collected onto charged slides (Superfrost; Colorado Histo-Prep, Fort Collins, CO), and one slide of each tissue was stained with hematoxylin and eosin (H & E) for microscopic examination. Tissues were scored using the following criteria: 0 = no apparent pathology/change, 1 = minimal change (minimally increased numbers of small lymphocytes, plasma cells, macrophages, and/or mast cells), 2 = mild change (mild inflammation, edema, and/or parafollicular expansion, secondary follicle formation, and presence of tingible body macrophages within lymph nodes), 3 = moderate change (as previously described, but more moderately extensive), 4 = marked changes (as previously described, but with severe inflammation, edema, and/or lymphoid reactivity)

### Quantification of IgA and IgG antibodies in saliva

Saliva from FIV-infected and sham-inoculated cats was evaluated for circulating antibodies at days 22, 36, 57, and 71 post-inoculation. Total IgA and IgG from saliva were quantified using microsphere immunoassay (MIA) protocols involving conjugation of magnetic microspheres with IgA or IgG capture antibodies [60]. Following conjugation protocols, a hemocytometer was used to determine microsphere concentrations and protein coupling was confirmed via incubation of microspheres with primary antibodies and/or PE-conjugated detection antibodies [51]. Successful coupling of antibody to microspheres was determined by a median fluorescence intensity (MFI) of >2,000. Saliva samples from FIV-infected and negative control cats were diluted 1:100 and 1:1000 in assay buffer for detection of IgG and IgA, respectively. These samples were then incubated in duplicate with approximately 2,500 conjugated beads per well in untreated, round-bottom 96-well plates [51, 60]. Total IgG and IgA antibody concentrations were calculated from an 8-point standard curve (2-fold dilution series, run in duplicate) using the MFI obtained from ≥100 microspheres per analyte per well (Bio-Plex^™^ Manager 5.0). Reagent concentrations, volumes, incubation times, acceptable standard recovery, and data analysis were performed as previously described [51, 60].

FIV-specific antibodies were detected using microsphere immunoassay (MIA) protocols involving conjugation of magnetic microspheres with FIV-specific capsid (CA) or envelope surface glycoprotein (SU_C36_-Fc) recombinant proteins [51, 60]. Following conjugation protocols, a hemocytometer was used to determine microsphere concentrations, and protein coupling was confirmed via incubation of microspheres with primary antibodies and/or PE-conjugated detection antibodies [51]. Successful coupling was determined by a median fluorescence intensity (MFI) of >2,000. All saliva samples from FIV-infected and negative control cats were diluted 1:10 in assay buffer and then incubated in duplicate with approximately 2,500 conjugated beads per well. All samples were assayed in conjunction with FIV-C and naïve reference samples diluted 1:50 in assay buffer, as well as four diluent control wells per experiment. The MFI was calculated from ≥100 microspheres per analyte per well (Bio-Plex^™^ Manager 5.0) and then used for data analysis. All reagent concentrations, volumes, incubation times, acceptable standard recovery, and data analysis were as previously described [51, 60].

### *In vitro* salivary inhibition of viral infection

Approximately 80μl of saliva was obtained from each of 6 healthy, non-infected SPF cats, and was pooled and used in same day *in vitro* experiments. Duplicate cell cultures consisting of GFox cells (CrFK cells overexpressing CD134) were established in 24-well plates at 180,000 cells/well and allowed to attach at 37°C overnight [53, 56]. GFox cell cultures were grown at 37°C in 5% CO_2_ in culture medium composed of Dulbecco's modified Eagle's medium (DMEM) with GlutaMAX-1, 10% fetal bovine serum (FBS), and 1x penicillin-streptomycin (10,000 U/liter penicillin and 10,000 μg/liter streptomycin), as well as 1 μg/ml of Fungizone^®^ (Amphotericin B; Life Technologies) [57]. At day 0, 50,000 TCID_50_ of FIV_C36_ was incubated with varying dilutions of saliva (1:100, 1:50, or no saliva (positive control)) in 1 ml of fresh culture media for 1 hour at 37°C [61]. Following incubation, infected media was pipetted onto Gfox cells plated in 1 ml of culture media, bringing the total volume in each well to 2 ml. Cell cultures were then incubated at 37°C for 12 hours, at which point all culture media was removed from each well, discarded, and replaced with 2ml of fresh culture media. At days 4, 6, 8 and 10 post-inoculation, 1ml of supernatant was removed from each well, frozen at -80°C, and replaced with 1 ml of fresh culture media. The supernatant collected from each well and each time point was then assayed for the detection of FIV p26 antigen by capture ELISA, measured at an absorbance of 450nm in 96-well flat bottom plates as previously described [58]. Percent inhibition was calculated from mean absorbance values (Abs) using the formula [(*X* − *Y*)/*X*] × 100, where *X* is fraction of cells infected in the absence of saliva (positive control) and *Y* is the fraction of cells infected in the presence of the saliva (1:100 or 1:50 saliva) [62]. For each plate, a parallel set of wells containing cells without virus was incubated with corresponding dilutions of saliva (1:100, 1:50, or no saliva (negative control)). GFox cells were visually inspected at days 4, 6, 8, and 10 post-inoculation by inverted light microscopy for evidence of cell growth, attachment, syncytial cell formation, detachment, and cell death.

### Statistical analyses

Kruskal–Wallis test, Pearson correlations, ANOVA, and repeated-measures ANOVA (RM-ANOVA) were used to compare differences in salivary viral and/or proviral load among FIV-infected individuals, between sample type (saliva, plasma/PBMC), for each tissue individually, and between tissue types (lymphoid versus mucosal). RM-ANOVA was used to assess antibody responses over time and treatment after log_10_-transformation. For *in vitro* experiments, RM-ANOVA with multiple comparisons was used to evaluate differences in mean absorbance values and percent inhibition among treatment groups over time. For all significant results, pair-wise comparisons were made by post-hoc analysis. Analyses were conducted in R (http://www.r-project.org/) or using GraphPad Prism 6.0 software (La Jolla, CA). P-values < 0.05 were considered significant.

## Results

### FIV viral RNA and proviral DNA is present in saliva of infected cats

**Fig 1:**
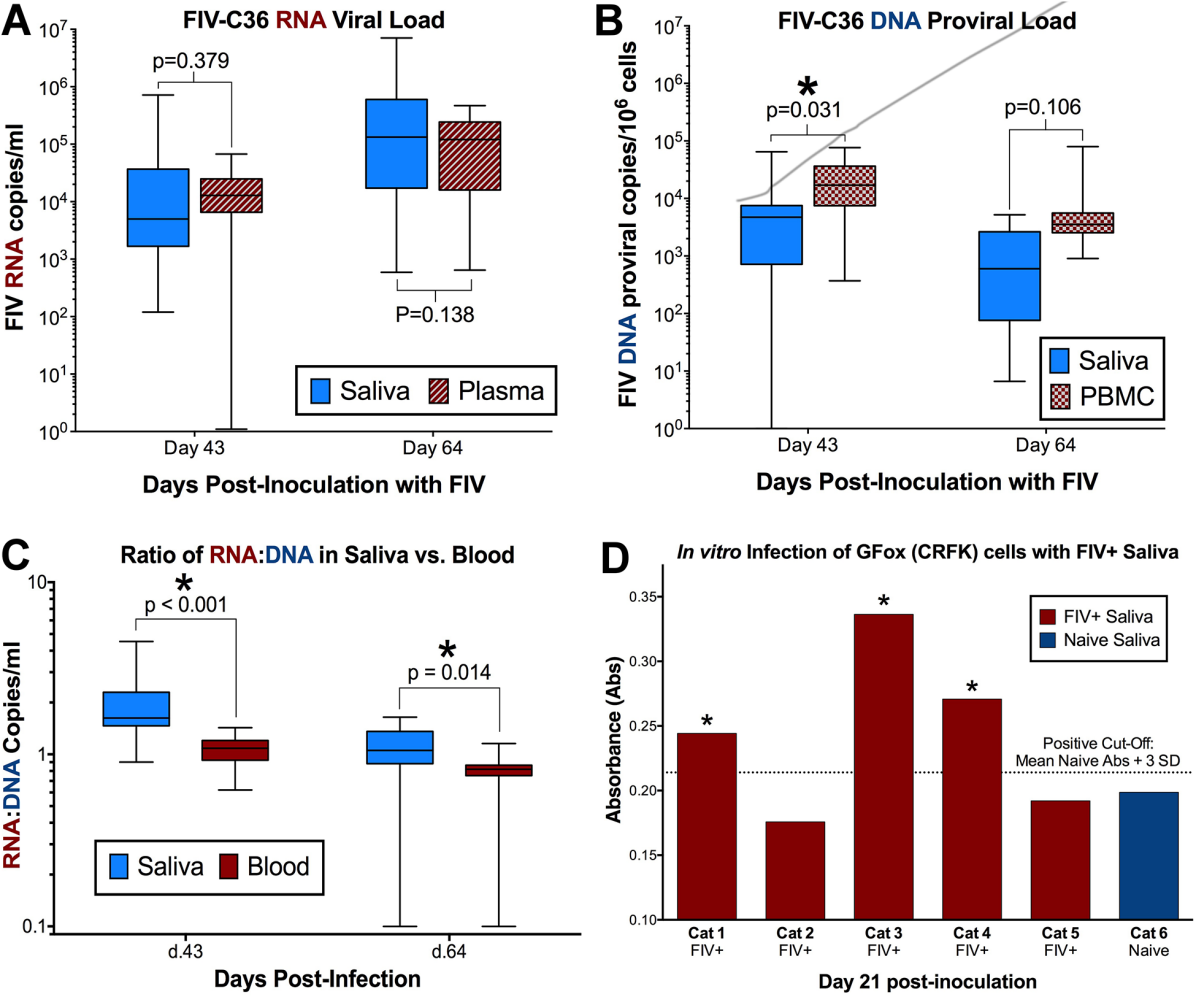
FIV viral RNA and proviral DNA are detected in saliva of infected cats. **(A)** Mean FIV viral RNA in saliva of infected cats is greater than observed in plasma (Mean: 446,000 copies/ml saliva, 85,100 copies/ml plasma). There is a trend (p = 0.165) for salivary viral load to be higher than plasma over the course of the study (RM-ANOVA). **(B)** Proviral DNA is present in saliva of infected cats, although levels tended to be 10-fold lower than circulating PBMC (p = 0.214; RM-ANOVA). **(C)** The ratio of FIV RNA to DNA is significantly higher in saliva than circulating levels in blood over the entire study (p = 0.036) and at each time point measured (day 43, p = <0.001; day 64, p = 0.014; RM-ANOVA with multiple comparisons). **(D)** Saliva from FIV-infected cats contains infectious FIV virus, as evidenced by FIV replication in GFox (CRFK) cells and the production of FIV viral particles following inoculation of saliva (day 21 post-inoculation, positive Cutoff = Mean Naïve Abs + 3*SD).

Cats infected with FIVC36 experienced plasma viremia, CD4/CD8 inversion and other clinical signs typically associated with acute pathogenic FIV infection [50]. Quantitative PCR analysis of saliva from FIV-infected cats revealed levels of viral RNA equivalent to that of plasma, with a trend for salivary viral load to be higher than plasma viremia over time (source p = 0.119, interaction p = 0.165, Fig 1A). Although present in lower quantities, saliva from FIV-infected cats also contained detectable quantities of proviral DNA (Fig 1B). Concurrent analysis of housekeeping gene GAPDH in saliva indicated that cellular DNA was abundant in saliva, was used as a proxy to determine cell equivalents per ml of saliva (range 3.19x10^5^-8.72x10^6^ cells/ml), and allowed us to calculate proviral DNA copies per saliva cell equivalent. FIV DNA levels tended to be approximately 10-fold lower in saliva than in peripheral PBMCs, although this trend did not differ over time (source p = 0.024, interaction p = 0.214). Interestingly, when DNA proviral and RNA viral loads were normalized to the number of copies per 1ml of saliva, the ratio of FIV RNA to DNA in saliva of infected cats was significantly higher over time than in the peripheral circulation, suggesting the oral cavity is an important site for persistent and enhanced FIV replication relative to peripheral circulation (source p = 0.001, interaction p = 0.036, Fig 1C).

### FIV is recovered from saliva of infected cats

The presence of infectious virus in saliva was confirmed by FIV p26 ELISA of tissue culture supernatants collected from GFox cells incubated for 21 days with saliva from 5 FIV-infected cats collected at 28 days post-FIV-infection (Fig 1D) [56]. Cells from infected cultures also exhibited characteristic cytopathic effect at this timepoint. This collection point correlated with high plasma viremia in FIV-infected cats [50], and confirms results reported by Matteucci et al. in 1993 [45] that infectious FIV is present in saliva of infected cats. FIV-negative saliva collected from sham-inoculated (naïve) cat at the same timepoint was included as a negative control.

### FIV viral and proviral loads are higher in oral lymphoid tissues

**Fig 2:**
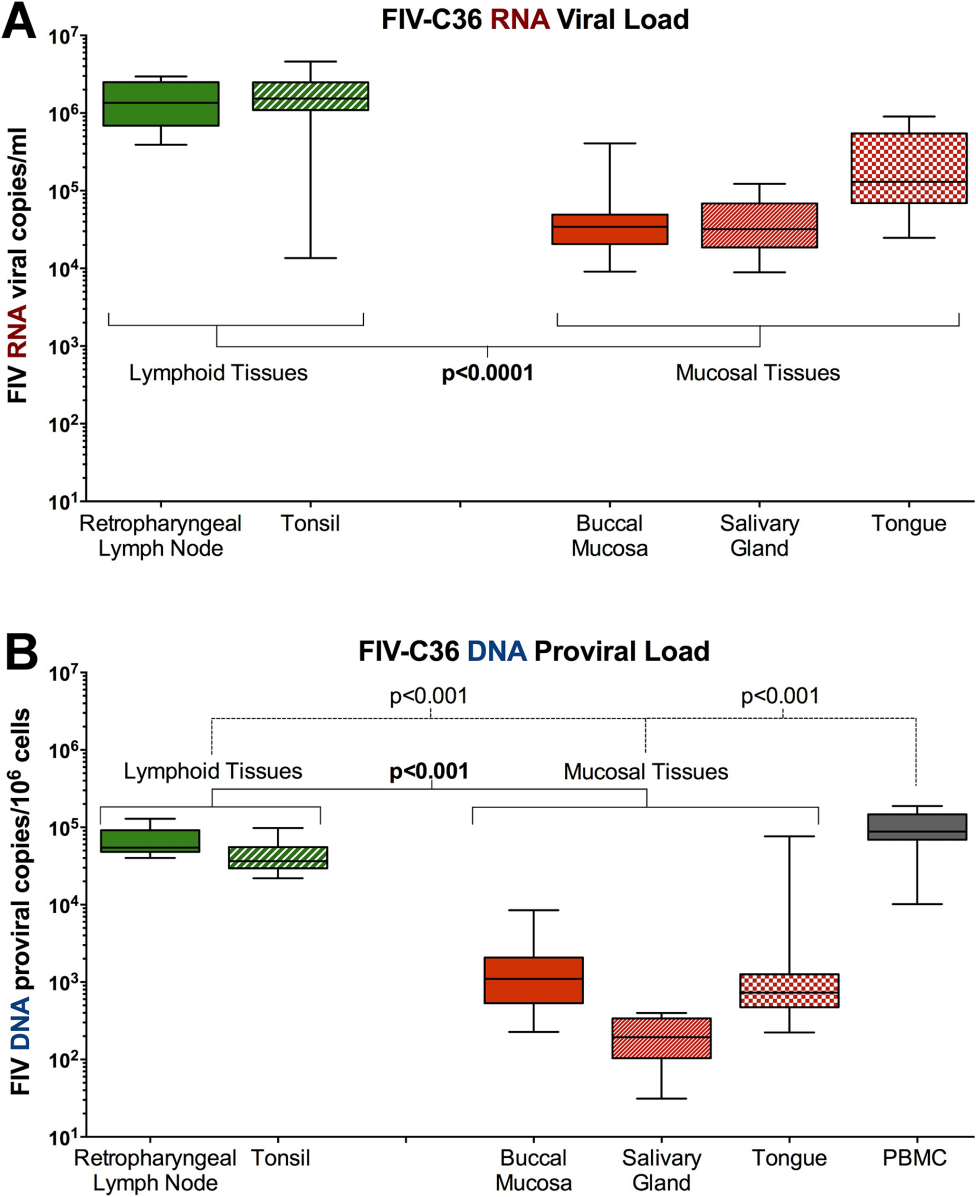
FIV RNA and DNA in oral lymphoid tissues is significantly higher than levels in non-lymphoid oral tissues. **(A)** FIV *RNA* levels in oral lymphoid tissues (retropharyngeal LN and tonsil) are significantly higher than non-lymphoid oral tissues (tongue, buccal mucosa, salivary gland) (p<0.0001), suggesting that oral lymphoid tissues serve as the site of viral replication and release into saliva. **(B)** FIV *proviral DNA* levels in oral lymphoid tissues are not as high as in circulating PBMC, but are significantly greater than in non-lymphoid tissues (p<0.0001), indicating that lymphoid organs may serve as oral reservoirs of FIV latency and persistence.

The retropharyngeal lymph node, tonsil, tongue, buccal mucosa, and salivary gland of FIV-infected cats contained appreciable quantities of viral RNA (Fig 2A). FIV RNA present within oral lymphoid tissues (retropharyngeal lymph node, tonsil) was significantly higher than oral mucosal tissues (p<0.001; tongue, buccal mucosa, salivary gland). Proviral DNA was also detected in all oral tissues, and oral lymphoid tissues contained significantly more FIV DNA than oral mucosal tissues (p<0.001) (Fig 2B), however, the quantity of FIV DNA in oral lymphoid tissues and mucosal tissues was lower than in circulating PBMC (p<0.001). Viral RNA and proviral DNA were not detected in the saliva or oral tissues from naïve animals (data not shown).

### FIV infection induces pathological changes in oral tissues

**Fig 3:**
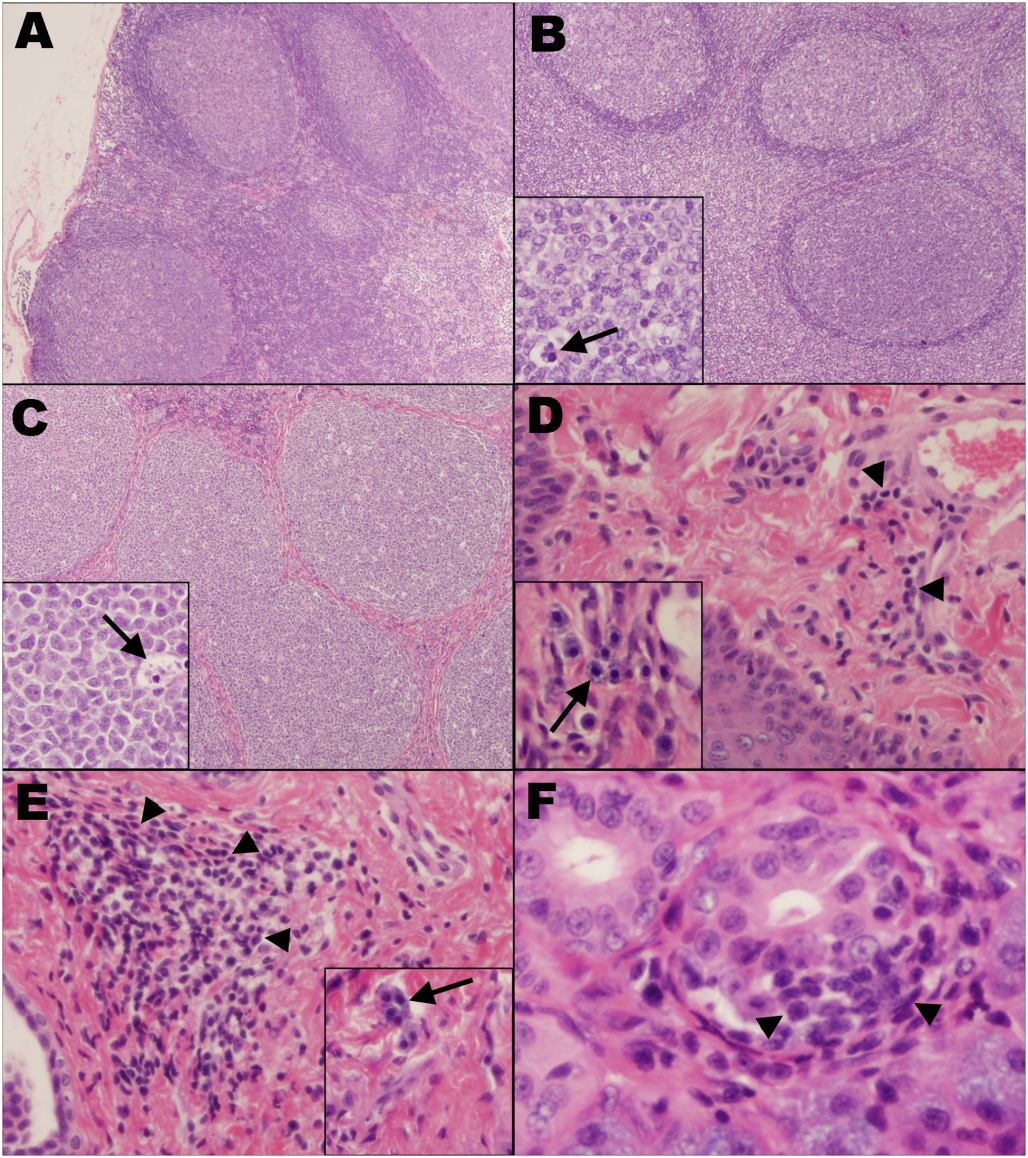
FIV induces mild to moderate pathology in oral tissues. **(A)** Retropharyngeal lymph node. Sham-inoculated control, HE stain, 40x. **(B)** Retropharyngeal lymph node and **(C)** palatine tonsil from FIV-infected cats exhibit moderate lymphoid hyperplasia with multifocally enlarged germinal centers and thin mantle zones, 40x. Higher magnification (insets, 200x) demonstrates tingible body macrophages (arrows). HE stain. The submucosa of the tongue **(D)** and buccal mucosa **(E)** are multifocally infiltrated by small to moderate numbers of small lymphocytes and plasma cells (arrows, 100x), as well as small numbers of scattered mast cells (arrows, inset, 200x). HE stain. **(F)** Submandibular salivary gland. Minimal numbers of small lymphocytes and plasma cells multifocally surround acini (arrowheads). HE stain, 400x.

**Fig 4:**
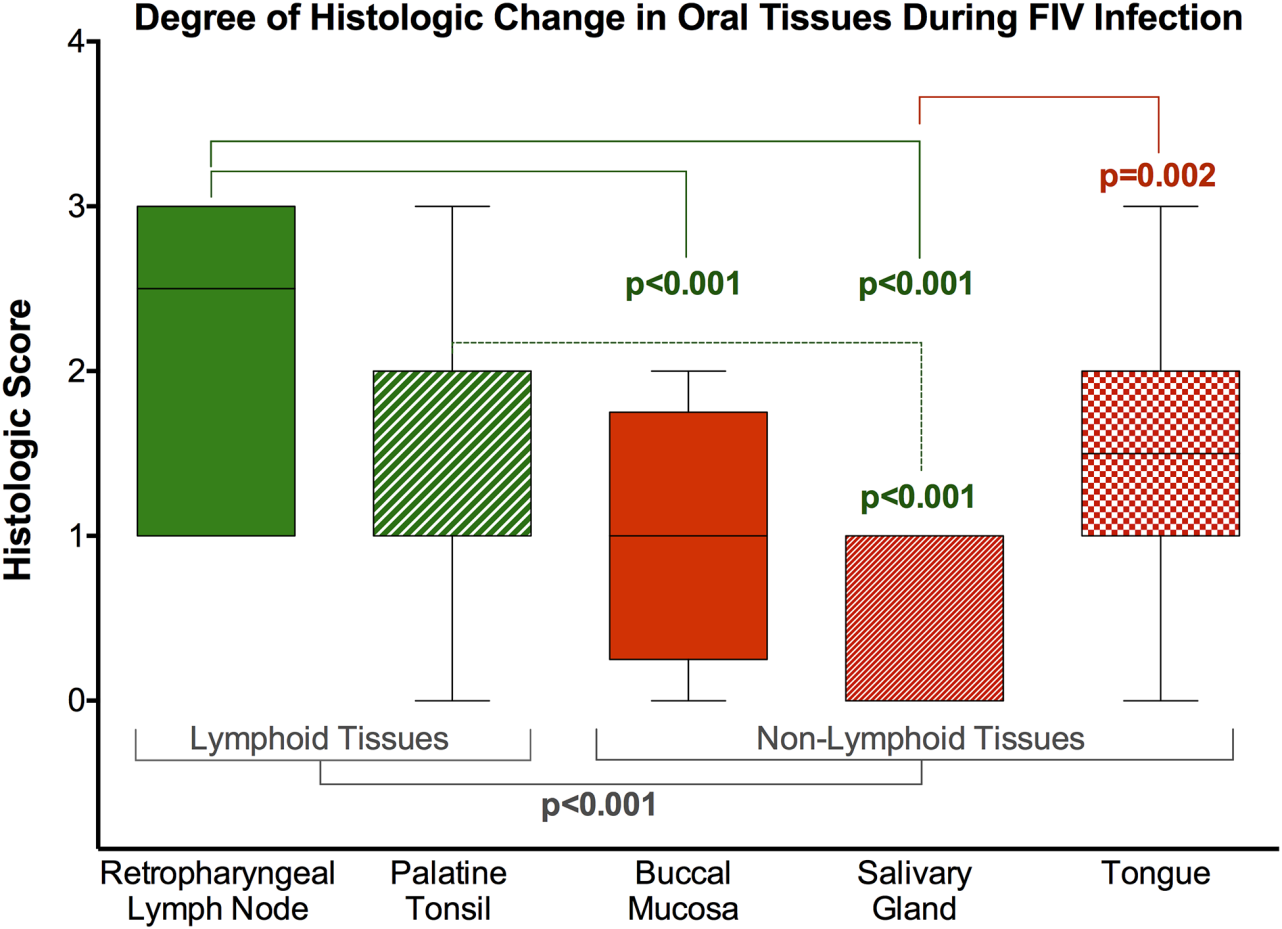
Oral lymphoid tissues exhibit a greater degree of histologic change than non-lymphoid tissues. The degree of histologic change in oral lymphoid tissues (retropharyngeal lymph node and tonsil) was significantly higher than in non-lymphoid tissues (tongue, buccal mucosa, and salivary gland) (p<0.001). The degree of histologic change was significantly greater in the retropharyngeal lymph node (p<0.001), palatine tonsil (p<0.001), and tongue (P = 0.002) than in the salivary gland. A greater degree of histologic change was also observed in the retropharyngeal lymph node compared to the buccal mucosa (p<0.001) (ANOVA with Tukey test for multiple comparisons).

All tissue sections from a sham-inoculated control animal were histologically unremarkable (Fig 3A and S1 Table). Of the 16 FIV-infected animals, the retropharyngeal lymph node (n = 10) (Fig 3B) and the palatine tonsil (n = 11) (Fig 3C) exhibited mild to moderate lymphoid hyperplasia, characterized by multifocal enlarged germinal centers with thin mantle zones and a frequent “starry-sky” appearance due to numerous tingible body macrophages. Eight FIV-infected individuals exhibited mild (n = 7) to moderate (n = 1) lymphoplasmacytic and histiocytic glossitis. In three of these animals, the submucosa was multifocally expanded by mild to moderate numbers of mast cells (Fig 3D). Additionally, 4 FIV-infected animals had a mild, multifocal, lymphoplasmacytic stomatitis of the buccal mucosa, with scattered mast cell infiltration observed in the same 3 animals in which the tongue was similarly affected (Fig 3E). Small numbers of scattered small lymphocytes and plasma cells were occasionally observed at the periphery and surrounding individual acini of the salivary glands in 7 of the FIV-infected cats (Fig 3F). Overall, the degree of histologic change in oral lymphoid tissues (retropharyngeal lymph node and tonsil) was significantly higher than in non-lymphoid tissues (tongue, buccal mucosa, and salivary gland; p<0.001, Fig 4). Individually, the degree of histologic change in the retropharyngeal lymph node (p<0.001), palatine tonsil (p<0.001), and tongue (p = 0.002) was significantly greater than in the salivary gland, and there were more histologic changes in the retropharyngeal lymph node compared to the buccal mucosa (p<0.001) (Fig 4).

### FIV-specific IgG increases in saliva of FIV+ cats, while IgA remains static

**Fig 5:**
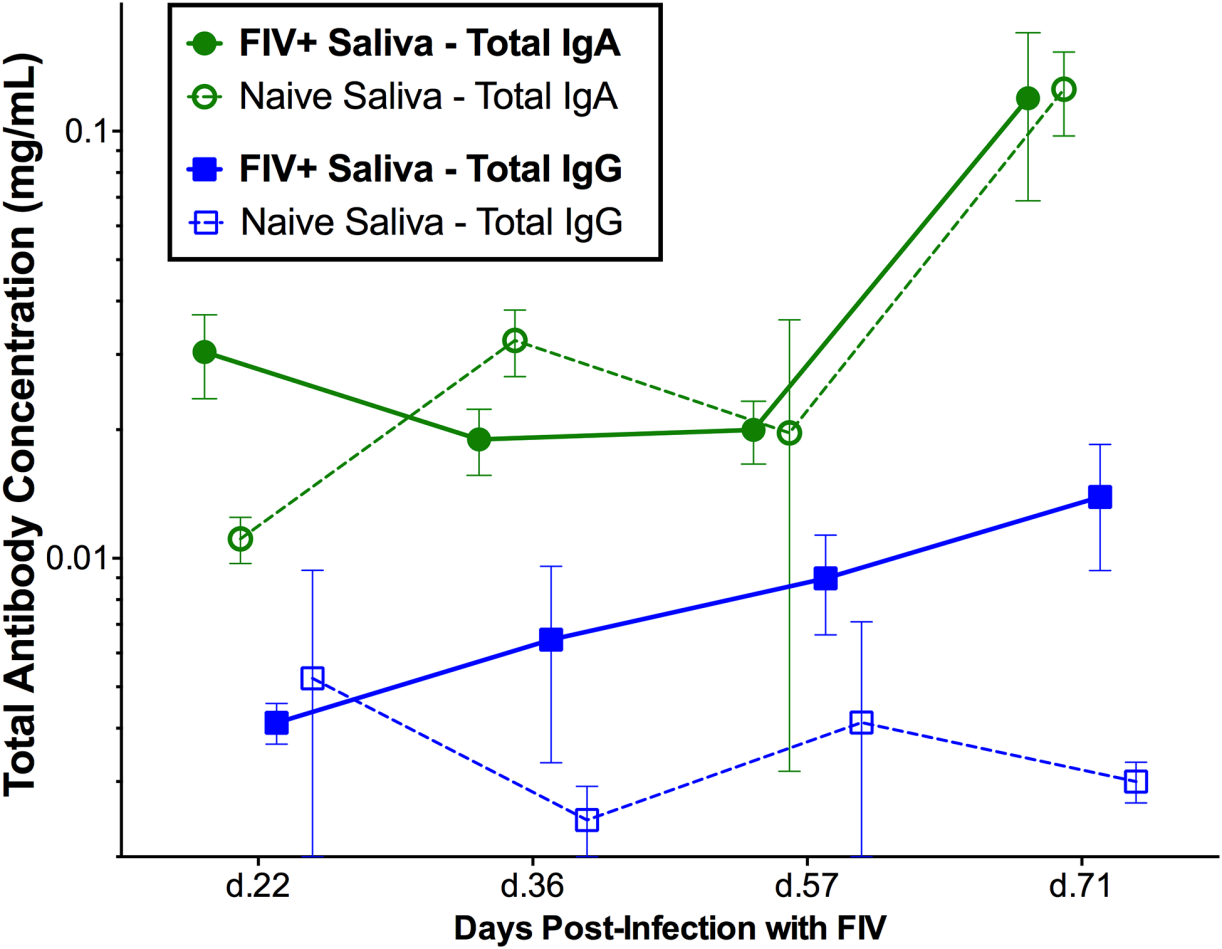
Limited IgA mucosal antibody response during FIV infection. There is a trend for salivary IgG concentrations to be slightly increased in FIV-infected cats following infection (solid blue line/squares) (mean ±SE; p = 0.216, RM-ANOVA). Salivary IgG remained constant in naïve animals (blue dashed line/clear squares). Salivary IgA concentrations did not differ significantly between FIV-infected cats (solid green line/circles) and naïve cats (dashed line/clear circles) over time (p = 0.969), but as expected, levels of IgA were higher in saliva than IgG.

**Fig 6:**
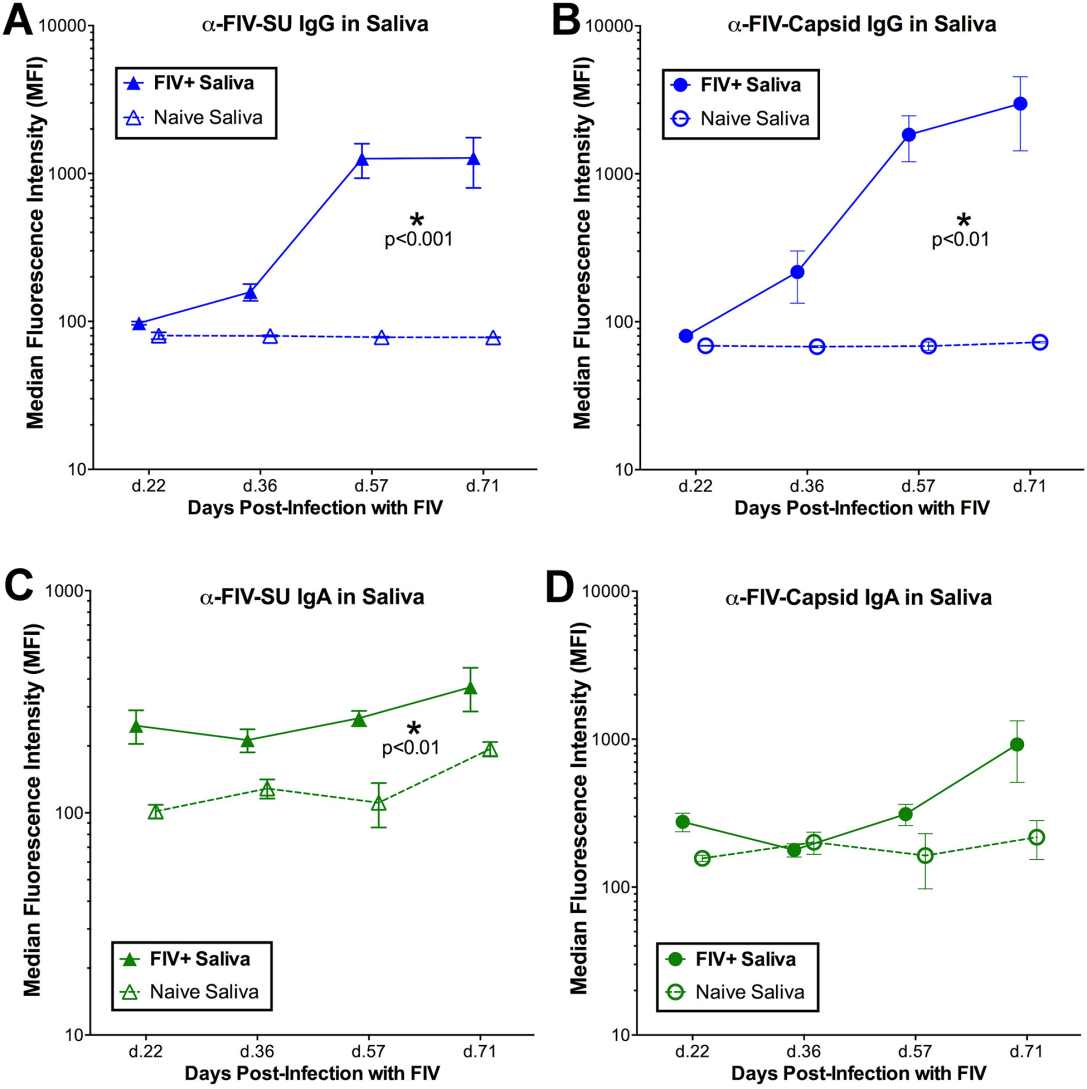
FIV specific IgG and IgA antibodies are detected in saliva of infected cats. **(A)** Anti-SU IgG and **(B)** anti-CA IgG antibody levels in saliva of FIV infected cats (solid blue lines) were significantly elevated (mean ±SE; treatment p<0.01 and p<0.001, respectively) compared to naïve animals (dotted blue lines) and increased significantly over time compared to naïve saliva (interaction anti-CA: p<0.05; anti-SU: p<0.001; RM-ANOVA). **(C)** Anti-SU IgA antibody levels in saliva of FIV infected cats (solid green lines) were significantly elevated compared to naïve animals (dotted green lines) (treatment p<0.01), but did not increase significantly over time compared to naïve saliva (interaction p = 0.569; RM-ANOVA). **(D)** No difference in salivary anti-CA IgA antibody levels was observed between FIV-positive animals and naïve animals.

Saliva samples were collected from FIV-infected and negative control cats at four time points, and total IgA and IgG antibodies from saliva were quantified by microsphere immunoassay (MIA). The concentration of total IgG in FIV-infected cat saliva ranged from 0.001 to 0.037mg/ml over the course of infection, while the total IgA concentration in FIV-infected cat saliva ranged from 0.005 to 0.026mg/ml. There was a slight trend for total salivary IgG to be increased in FIV-infected cats compared to naive individuals (treatment p = 0.216), however, total IgA antibody levels did not differ in saliva from FIV-infected and sham-inoculated cats (treatment p = 0.999) (Fig 5). FIV-specific anti-SU and anti-CA IgG antibodies were consistently detected in saliva of FIV-infected cats and increased significantly over time (anti-SU interaction p<0.001; anti-CA interaction p<0.05, Fig 6A and 6B). Although anti-SU IgA antibodies were elevated in saliva from FIV-infected cats (treatment p<0.05, Fig 6C), they did not increase over time compared to the background of uninfected saliva (interaction p = 0.569). Furthermore, anti-CA IgA antibodies were not significantly elevated in FIV-infected saliva (treatment p = 0.809, Fig 6D).

### Saliva exhibits an inhibitory effect on FIV infection *in vitro*

**Fig 7:**
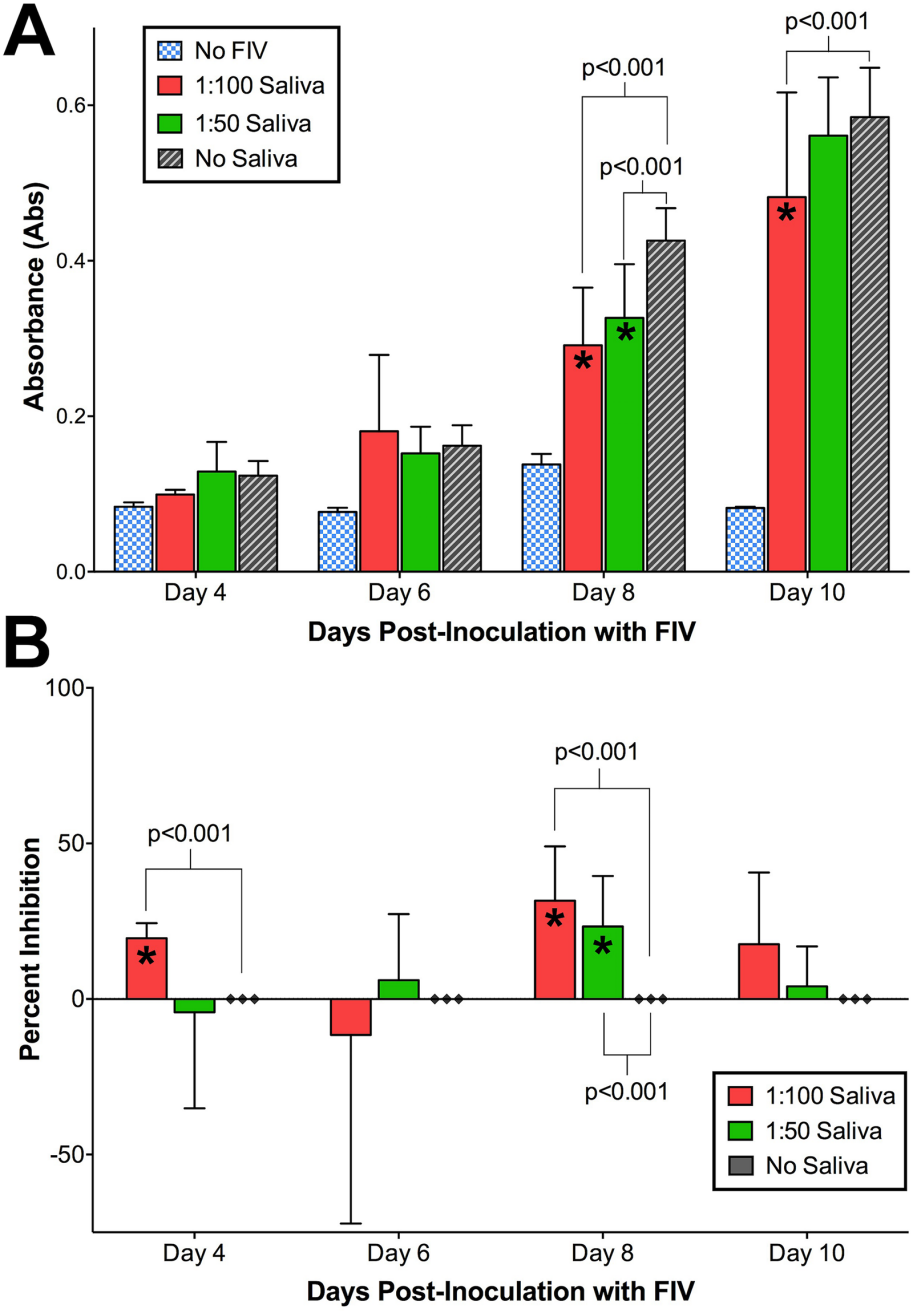
Naïve cat saliva inhibits FIV replication. FIV_C36_ was incubated with 1:50 or 1:100 dilutions of naïve cat saliva in duplicate and inoculated onto CRFK cultures as described in the text. A ‘No Saliva’ virus-only positive control represented 100% FIV growth as measured by ELISA absorbance. **(A)** Mean ELISA absorbance values increased for all treatments except for virus-negative control (blue-checkered bars). Absorbance values for FIV pre-incubated with saliva at both 1:100 and 1:50 dilutions were significantly lower (p<0.001; RM-ANOVA) than the no saliva control (gray-striped bars), indicating a lower FIV replication rate in the presence of saliva. **(B)** Analysis of percent inhibition over time revealed a significant inhibitory effect with saliva treatments differing over time (p<0.05; RM-ANOVA) and at individual time points (days 4 and 8 post-inoculation) relative to the ‘No Saliva’ virus-only positive controls.

Naïve cat saliva significantly inhibited FIV_C36_ growth in GFox cells (CrFK cells overexpressing feline receptor CD134) over time, indicated by lower FIV p26 ELISA absorbance values in the presence of saliva (interaction p<0.001, Fig 7A). Percent viral inhibition compared to no-saliva controls differed significantly over time, peaking at 8 days post-inoculation and declining slightly thereafter (interaction p<0.05, Fig 7B).

## Discussion

Our findings document that: (1) FIV infection predominates in oral lymphoid tissues versus mucosal and salivary gland, resulting in mild to moderate histologic pathology (Figs 2–4, S1 Table), and provide evidence of a site of viral persistence and chronic immune dysregulation; (2) FIV viral RNA ratio to proviral load, a surrogate for viral replication rate, is significantly higher in saliva than in plasma/circulating PBMC (Fig 1C), suggesting enhanced FIV replication occurs at a site where viral shedding is thought to take place; (3) though FIV-specific IgG antibodies increase in saliva during infection, secretion of FIV-specific IgA in saliva is impaired (Figs 5 and 6), suggesting one potential mechanism for chronic oral infection, the development of opportunistic oral disease, and transmission of FIV via oral secretions; and, (4) saliva from uninfected SPF cats harbors *in vitro* anti-viral properties (Fig 7), which may be an important mechanism for limiting ‘passive’ FIV transmissions among FIV-infected and uninfected cats in stable social structures.

**Fig 8:**
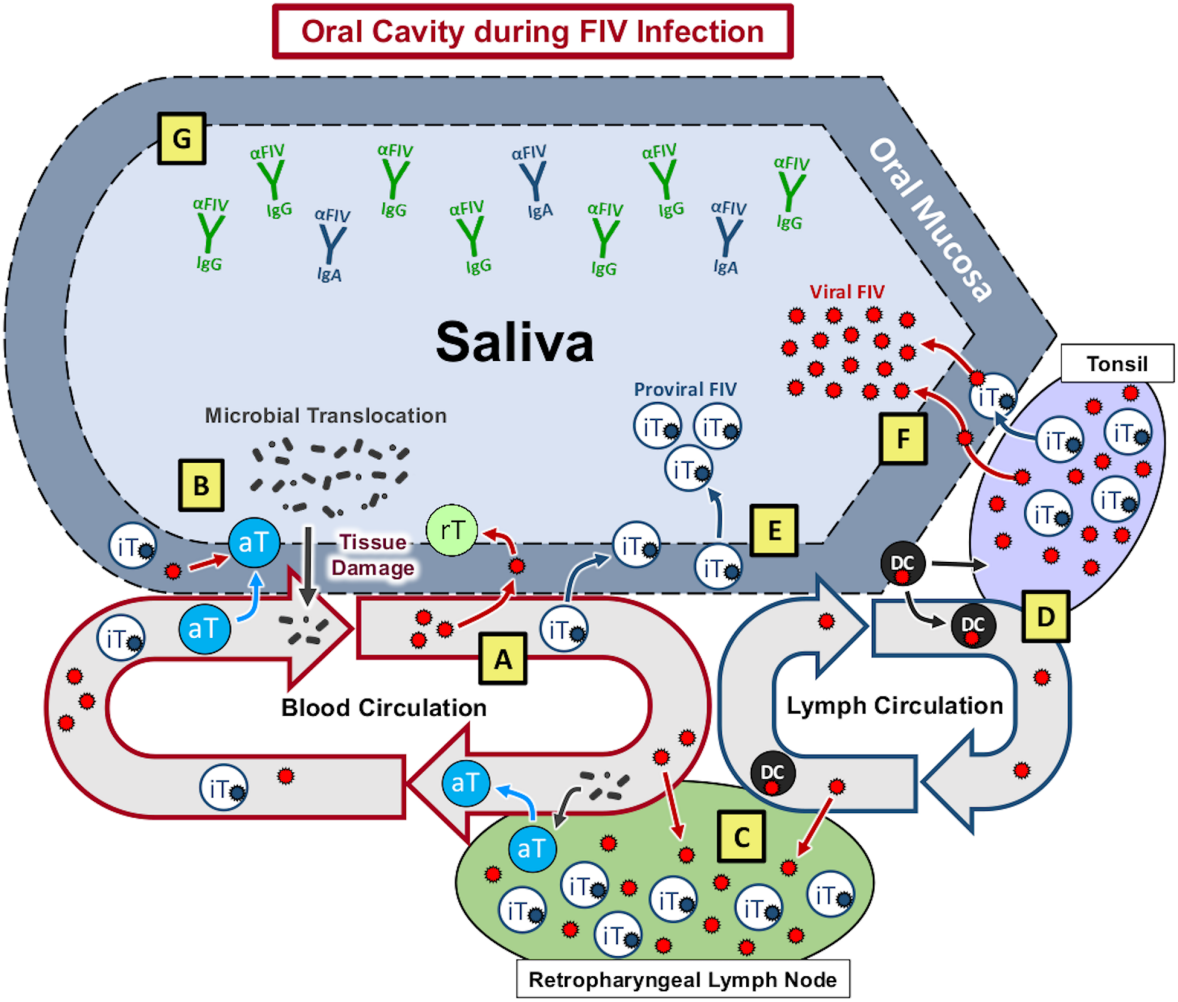
Proposed model of oral FIV pathogenesis. Similar to the gastrointestinal tract, FIV exhibits a tropism for oral tissues, providing a pathway for circulating virus to extravasate and infect resting T-lymphocytes (rT) and dendritic cells (DC). **(A)** As documented in the intestinal mucosa, infection and depletion of resting mucosal lymphocytes in the oral mucosa may cause inflammation, damage mucosal barriers, and subsequent translocation of oral microbes; resulting in lymphocyte activation and recruitment to oral mucosal tissues. **(B)** Mucosal injury initiates a chronic cycle of immune activation and provides a renewable source for target cell infection by recruiting susceptible cells to the site of injury. Peripheral FIV-infected cells traffic to oral lymphoid tissue via lymphatics **(C)** or direct migration to tonsils **(D)** resulting in antigen presentation, T-lymphocyte activation (aT), and infection of resident leukocytes, establishing a reservoir of persistent FIV replication in latently infected T-lymphocytes (iT) in oral lymphoid tissues. **(E)** FIV infected cells are likely shed from oral mucosal sites into saliva, resulting in the presence of salivary proviral DNA. Cells within oral lymphoid tissues have enhanced FIV replicative activity, resulting in a higher ratio of FIV viral RNA to proviral DNA than noted in the peripheral circulation. **(F)** FIV RNA in saliva may be achieved by direct release of virus particles from infected cells into saliva via the intimate association of tonsillar lymphoid tissue with the oral mucosa. **(G)** While FIV specific IgG responses are detected in saliva, anti-FIV IgA antibodies are not specifically enhanced, allowing FIV virus and infected cells to persist in saliva at high levels.

Collectively, these data provide a descriptive analysis of viral, immunological, and pathogenic features of oral FIV infection, and highlight a continuum between salivary and peripheral viral kinetics that is represented in Fig 8. Initial events of FIV infection occur systemically, resulting in peripheral lymphocyte infection, viremia, and an anti-FIV antibody response in which IgG antibodies predominate over IgA [39, 51, 52, 63]. During HIV and SIV infection, CD4+ T-cells are rapidly infected and severely depleted from the intestinal mucosal surface, resulting in loss of mucosal integrity, reduced capacity to control potential pathogens at mucosal surfaces, and subsequent triggering of pro-inflammatory responses [25, 26, 28, 29, 31, 32]. Comparatively, we demonstrate that FIV exhibits a significant tropism for oral lymphoid tissues, which may potentially serve as initial sites for circulating virus to infect resting T-lymphocytes and dendritic cells (Fig 8A). As an extension of the digestive tract, similar effects of viral-induced immunosuppression may likely occur at the oral mucosa, resulting in a chronic cycle of lymphocyte depletion, microbial translocation, immune stimulation, and leukocyte recruitment and consequent infection (Fig 8B), as evidenced by FIV accumulation and histologic changes in oral tissues.

FIV virus particles and/or infected cells traffic to oral lymphoid tissues via circulation (Fig 8C) or by direct migration to the tonsils from the oral mucosa (Fig 8D), subsequently infecting resident leukocytes and establishing a reservoir of persistent FIV replication in latently infected T-lymphocytes within oral lymphoid tissues. Viral RNA and proviral DNA are also detected in non-lymphoid oral tissues, but at a much lower quantity than in lymphoid tissues, indicating that these sites (tongue, buccal mucosa, salivary gland) are not a primary site of FIV persistence. However, accumulation of proviral DNA in these tissues suggests that passive migration of FIV-infected cells may occur from the periphery to non-lymphoid oral tissues, which may then perpetuate FIV-infection at these sites.

Although detectable, the quantity of FIV DNA is lower in saliva than in circulating PBMCs, and reflects the concept that the FIV-infected cells being shed into saliva may be shed via the oral mucosal epithelium in lieu of oral lymphoid reservoirs (Fig 8E), which contain larger amounts of FIV proviral DNA. In contrast, FIV RNA appears later in saliva than in plasma [63], but salivary FIV RNA concentration is equivalent to plasma, and FIV RNA is present in high quantities in oral lymphoid tissues (Fig 1). Our finding that FIV RNA is proportionally higher (compared to proviral load) in saliva than in plasma suggests that the oral cavity is a preferential site for viral RNA transcription. The observed predilection of FIV for oral lymphoid tissues indicates that these tissues may serve as an important reservoir and primary source of viral replication and shedding of viral RNA into the saliva (Fig 8F). Systemic hyperplasia of lymphoid structures is a prominent feature of FIV infection, and the histologic changes observed support an analogous impact on oral lymphoid tissues; thus supplying an ample source of target cells for persistent FIV replication [39, 64–66]. Moreover, the anatomic distribution of the palatine, paraepiglottic, pharyngeal and lingual tonsils provides an intimate association with the overlying oral mucosa, and highlights the potential for these organs to serve as sites of extrusion of FIV into saliva through trafficking of infected cells across the adjacent mucosal epithelium [67].

IgA represents the primary mucosal antibody that limits numbers of mucosa-associated bacteria and prevents bacterial penetration of host tissues. This is best demonstrated by IgA deficiency, which results in increased penetration of symbiotic bacteria into the host tissues and consequent inflammation [68–71]. In the oral cavity and gastrointestinal tract, differentiated plasma cells secrete IgA, which then transcytoses across the epithelial layer to the apical surface of the epithelium where it works to crucially maintain homeostasis through luminal compartmentalization of intestinal bacteria [68]. However, during FIV infection, IgG antibodies increase in saliva and in the peripheral circulation of infected cats [51], but the IgA antibody response remains static at both of these sites. Furthermore, FIV-specific IgA antibodies against capsid are undetectable in saliva, and anti-SU IgA does not increase over time. In contrast, both anti-SU and anti-CA IgG antibodies increase significantly in saliva during FIV infection (Figs 6B and 8G). These results indicate a failure to mount an effective mucosal antibody response during FIV infection, which may perpetuate oral mucosal viral infection as well as changes in the oral microbiome, thus contributing to microbial translocation and a cycle of immune dysfunction.

Currently, FIV infection is diagnosed clinically by either lateral-flow serum ELISA or microwell, and western blot and immunofluorescent antibody (IFA) assays are frequently used as confirmatory tests following positive ELISA results [72]. Unfortunately, these assays require blood samples to screen for serum FIV-specific antibodies, which may be impractical to acquire in intractable patients, or during field operations to capture and control the feral cat population. The presence of anti-FIV IgG antibodies in saliva suggests that a reliable alternative diagnostic assay can be developed based upon saliva sampling, and may allow for methods to differentiate between vaccinated and un-vaccinated cats as previously suggested by Wood et al. [51]. Microsphere immunoassay technology or other sensitive antibody detection assays may be of great use in clinical applications to test for FIV in a less invasive manner, as these assays only require passage of an oral swab along the interior of the oral cavity. Furthermore, optimization to detect FIV in saliva may be directly adapted to test human saliva for HIV without the need for blood sampling, thus increasing the capacity to screen large numbers of patients in endemic areas.

Results of this study demonstrate that uninfected feline saliva contains a component with anti-FIV properties, a feature that may have implication for FIV transmission in natural settings. Prolonged contact between animals is typically required for transmission of FIV, and feral male cats are most at risk for FIV infection. Numerous studies have indicated that horizontal transmission through casual contact such as grooming is extremely inefficient and rarely occurs in the absence of biting, i.e. amongst cats maintained indoors or in stable groups [47, 73–75]. Our findings suggest that cats which groom each other may be less susceptible to FIV transmission via fomites and cat to cat oral contact because of unidentified innate factors in saliva, which inhibit infection via the oral route. While experimental transmission studies have documented potential for oral transmission by inoculation of virus into the oral cavity [76, 77], these studies provided an artificially high dose of concentrated virus, which would be an unusual situation to encounter in a natural transmission setting.

Previous studies in humans infected with HIV have demonstrated that significant anti-viral activity in human saliva is conferred by numerous cofactors and immune modulators, such as anti-HIV antibodies, defensins, thrombospondin-1, proline-rich proteins, salivary agglutinin, and secretory leukocyte protease inhibitor (SLPI) [14, 15]. It is possible that naïve feline saliva may confer anti-viral activity *in* vivo by similar mechanisms and effector molecules. However, the capacity of FIV to be transmitted via saliva despite its potential inhibitory properties may indicate alterations in the composition of saliva in infected cats, or perhaps other viral-induced mechanisms by which FIV may overcome these inhibitory effects. Further investigation is warranted to determine whether feline saliva contains inhibitory molecules similar to those in human saliva and how the composition of feline saliva changes following FIV infection.

Although HIV transmission typically occurs via parenteral or transmucosal venereal routes, recent epidemiological studies have provided definitive evidence that HIV can be transmitted by receptive oral intercourse, and occasional cases of transmission by biting have been documented [7–13]. Furthermore, significant quantities of both HIV viral RNA and proviral DNA have been detected in saliva of infected individuals, with significant correlations between salivary and plasma RNA levels [16, 17]. Oral HIV transmission occurs less frequently than in FIV transmission, likely due to the absence of aggressive biting in human populations versus cat populations [7, 11, 21, 78]. The use of SIV animal models has provided further proof that oral transmission of primate lentiviral infections is possible, and experimental SIV studies have helped to understand the oral immune response during infection [13, 79–81]. Moreover, retroviral-induced oral disease continues to affect a high proportion of individuals despite the success of highly active antiretroviral therapy (HAART), and is a common manifestation of both HIV and FIV infection [38, 41–43, 76]. Oral lesions are not typically observed during SIV infection and may highlight significant limitations of the SIV model to study HIV-induced oral disease [82, 83]. In contrast, FIV produces oral lesions in cats that closely resemble Linear Gingival Erythema and Necrotizing Ulcerative Gingivitis in humans with HIV-induced disease, and opportunistic microorganisms detected in saliva of HIV-positive individuals (*Candida albicans*, *Fusobacterium sp*., *Streptococcus sp*., *Prevotella sp*., *Campylobacter sp*., and *Porphyromonas gingivalis*) are also implicated in feline oral disease [1, 6, 31, 38, 39, 41–43, 47, 84–88]. Indeed, shifts in oral microbial structure during HIV and FIV infection have been increasingly linked to disease phenotypes, and the use of a feline animal model is a pragmatic solution to assess the impact of novel therapeutic strategies [1, 25, 38, 41, 84, 86, 89, 90]. An effective anti-HIV vaccine or prophylactic therapeutic agent that will cure or prevent HIV infection will likely need to function at the mucosal surface, and FIV provides a relevant animal model that might be exploited to specifically assess oral mucosal lentiviral disease and interventions [13, 91, 92].

The results of this study provide new evidence for unique pathogenic features of oral FIV infection and suggest mechanisms that overcome host resistance. Manipulation of constituents of saliva, inhibition of viral replication at oral lymphoid sites, or enhancement of anti-viral IgA production may represent novel therapeutic interventions to reduce or eliminate oral FIV infection, as well as the potential to treat HIV-induced oral disease. Our results additionally provide a plausible hypothesis linking site-specific viral replication, mucosal immune deficiency, and salivary inhibition to the natural transmission and infection cycle of FIV.

## Supporting information

S1 Table Histopathologic changes are observed in FIV-infected cats. Histologic scores of oral tissues from FIV positive animals on a scale of 0–4 (described in text). Most prominent pathologic changes are demonstrated in Fig 3, and consist of moderate lymphoid hyperplasia in the retropharyngeal lymph node (RP LN) and tonsil, followed by mild to moderate glossitis and stomatitis in the tongue and buccal mucosa. Differences in the degree of histologic change between oral tissues are summarized in Fig 4.
(TIF)
