# Supplementary figures and images for: Pathogenesis of oral FIV infection
Source: PLoS One. 2017 Sep 21;12(9):e0185138. doi: 10.1371/journal.pone.0185138 (PMC5608358; doi:10.1371/journal.pone.0185138)

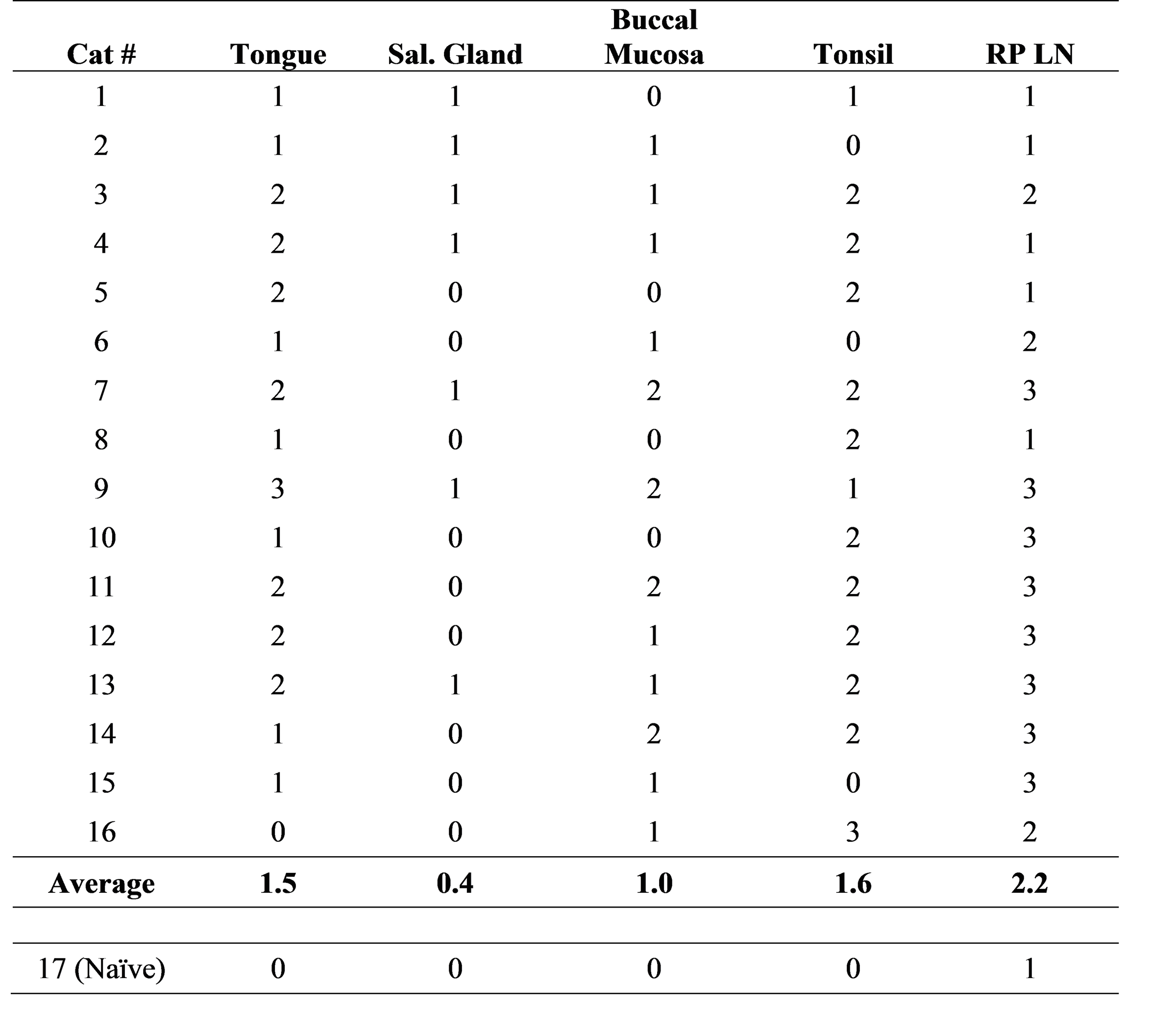

Supplement: S1 Table — Histologic scores of oral tissues from FIV positive animals on a scale of 0–4 (described in text). Most prominent pathologic changes are demonstrated in Fig 3, and consist of moderate lymphoid hyperplasia in the retropharyngeal lymph node (RP LN) and tonsil, followed by mild to moderate glossitis and stomatitis in the tongue and buccal mucosa. Differences in the degree of histologic change between oral tissues are summarized in Fig 4. (TIF) [file pone.0185138.s001.tif]
